# Beyond Diabetes: Continuous Glucose Monitoring as a Candidate Precision Tool for Cardiovascular Prevention and Healthy Longevity—A Hypothesis-Generating Narrative Review

**DOI:** 10.3390/medicina62081513

**Published:** 2026-08-06

**Authors:** Cristina Văcărescu, Dragos Cozma

**Affiliations:** 1 Institute of Cardiovascular Diseases Timisoara, 300310 Timisoara, Romania; dragos.cozma@umft.ro; 2 Cardiology Department, “Victor Babeș” University of Medicine and Pharmacy Timisoara, 300041 Timisoara, Romania; 3 Research Center of the Institute of Cardiovascular Diseases Timisoara, 300310 Timisoara, Romania

**Keywords:** continuous glucose monitoring, glycemic variability, longevity medicine, preventive cardiology, metformin, acarbose, intermittent fasting, cardiovascular prevention

## Abstract

*Background and Objectives:* Cardiovascular disease remains the leading cause of premature mortality worldwide. Subclinical glucose dysregulation, a contributor to accelerated vascular aging, is undetectable by conventional screening in apparently healthy individuals; even within the normal glycemic range, postprandial glucose excursions promote endothelial injury and inflammatory pathways independently of mean glucose levels. *Hypothesis:* Continuous glucose monitoring (CGM)-guided metabolic phenotyping, combined with personalized dietary optimization, structured fasting protocols, and selective longevity-oriented pharmacotherapy, constitutes a mechanistically coherent, hypothesis-generating preventive strategy that may attenuate cardiovascular risk and biological aging in apparently healthy non-diabetic adults, pending confirmation in prospective outcome trials. *Materials and Methods:* This narrative review synthesizes evidence from prospective cohort studies, randomized controlled trials, and mechanistic investigations connecting CGM-guided metabolic assessment with preventive cardiology and the emerging field of longevity medicine, focusing on glycemic variability biology, nutrient-sensing pathways, and the cardiovascular and longevity profiles of low-dose metformin and acarbose. *Results:* CGM-derived metrics capture inter-individual glycemic variability invisible to standard assessments and provide behavioral feedback for dietary personalization. Structured fasting and low-dose metformin converge on shared nutrient-sensing pathways implicated in both vascular aging and longevity, with CGM enabling objective confirmation of metabolic adaptation. Acarbose has shown cardiovascular and lifespan benefit signals in secondary trial analyses and preclinical longevity models, though these findings require replication and are not yet established in non-diabetic populations. *Conclusions:* We propose a four-phase research framework integrating CGM metabolic phenotyping, dietary optimization, fasting titration, and selective pharmacological augmentation for apparently healthy adults at cardiovascular risk. Prospective hard-endpoint trials are lacking, and this framework should be regarded as hypothesis-generating rather than an established clinical strategy, warranting rigorous outcome-based evaluation before clinical adoption.

## 1. Introduction

Cardiovascular disease (CVD) remains the leading cause of premature mortality worldwide, responsible for approximately 18 million deaths annually and generating economic costs exceeding €282 billion per year in the European region alone [1]. While traditional risk factor frameworks—hypertension, dyslipidemia, smoking, and overt diabetes—have guided primary prevention for decades, a growing body of evidence highlights the cardiovascular and longevity consequences of subclinical glucose dysregulation in individuals without manifest metabolic disease [2].

Even within the conventionally “normal” glycemic range, individuals exhibit highly divergent postprandial glycemic responses to identical meals—a phenomenon driven by differences in gut microbiome composition, dietary fiber intake, meal timing, physical activity patterns, and insulin secretory kinetics [3]. These inter-individual differences, invisible to HbA1c measurements and conventional fasting glucose assessments, are now measurable in real time by continuous glucose monitoring (CGM). Acute glycemic excursions generate oxidative stress, trigger endothelial dysfunction, promote advanced glycation endproduct (AGE) formation, and accelerate atherogenesis through mechanisms that operate independently of mean glucose levels [4].

Simultaneously, the biology of aging has been transformed by the discovery that nutrient-sensing pathways—the insulin/IGF-1 axis, mTORC1, AMPK, and sirtuins—are the principal molecular determinants of both metabolic health and organismal lifespan [5]. Interventions that modulate these pathways favorably, including caloric restriction, intermittent fasting, prolonged fasting, and specific pharmacological agents, produce consistent lifespan extension in model organisms and compelling cardiovascular benefit signals in human studies [6].

This review synthesizes the evidence connecting CGM-guided metabolic self-knowledge with preventive cardiology and longevity medicine. We argue that CGM provides not merely a diagnostic window, but an actionable behavioral feedback mechanism that, when combined with structured fasting protocols and longevity-oriented pharmacology (low-dose metformin and acarbose), constitutes a comprehensive preventive strategy accessible to apparently healthy individuals.

We wish to clarify the scope and organizing principle of this review at the outset. CGM is not treated here as an isolated diagnostic technology to be reviewed in isolation, but as the common monitoring and behavioral-feedback layer across a set of otherwise distinct interventions—dietary personalization, intermittent and prolonged fasting, and low-dose metformin and acarbose—that converge mechanistically on the same nutrient-sensing pathways discussed above. Fasting physiology and the pharmacology of metformin and acarbose are therefore discussed in this review specifically insofar as CGM data can inform patient selection, titrate intervention intensity, or verify metabolic response to these interventions, rather than as independently exhaustive treatments of fasting physiology or of each drug’s full pharmacology in their own right. This CGM-centered organizing principle is intended to justify the integration of these otherwise diverse topics within a single conceptual framework; readers seeking a comprehensive independent treatment of fasting biology or of metformin/acarbose pharmacology more broadly are referred to the dedicated literature on each topic, portions of which we cite throughout.

In this review, the term “longevity medicine” is used descriptively to refer to an emerging, non-standardized area of clinical interest concerned with extending healthspan and reducing age-related disease risk in apparently healthy individuals. It is not currently a formally recognized medical specialty, and it lacks standardized diagnostic criteria, validated clinical endpoints, or regulatory recognition. We use the term with this caveat throughout, and it should not be read as implying an established clinical discipline.

For clarity, this review uses the following terms with the specific meanings defined here. “Apparently healthy” refers to individuals without a diagnosed chronic disease at the time of assessment, though they may harbor unrecognized cardiometabolic risk factors; this is the broadest population discussed and is the primary population of interest for this review. “Non-diabetic” refers specifically to individuals who do not meet current diagnostic criteria for type 1 or type 2 diabetes mellitus, and may include individuals with normal glucose tolerance or prediabetes. “Prediabetes” refers to individuals meeting standard glycemic criteria for impaired fasting glucose, impaired glucose tolerance, or an intermediate HbA1c range, as defined by current diagnostic guidelines. “High-risk” is used to describe individuals with an elevated calculated cardiovascular or metabolic disease risk (e.g., via validated risk scores, family history, or established risk factors) and is not synonymous with a specific glycemic category; a high-risk individual may be normoglycemic, prediabetic, or non-diabetic. “Metabolically unhealthy” refers to the presence of one or more components of metabolic syndrome or related dysmetabolism, independent of diabetes status. Where a specific population is under discussion, we specify the applicable term(s) explicitly rather than using these terms interchangeably.

## 2. Materials and Methods

### 2.1. Search Strategy

This is a narrative review; it was not conducted according to a pre-registered systematic review protocol. Literature was identified through searches of PubMed/MEDLINE, Google Scholar, and Embase/Web of Science, conducted iteratively during manuscript preparation. Search terms combined concepts related to continuous glucose monitoring, glycemic variability, postprandial glucose, endothelial dysfunction, nutrient-sensing pathways (mTORC1, AMPK, SIRT1, insulin/IGF-1), fasting and caloric restriction, metformin, acarbose, cardiovascular prevention, and longevity/healthspan, combined using Boolean operators (e.g., “continuous glucose monitoring” AND “cardiovascular”; “metformin” AND “longevity”; “acarbose” AND “lifespan”). No formal date restriction was applied; searches prioritized landmark trials and foundational mechanistic studies regardless of publication date, supplemented by more recent literature (through early 2026) identifying evolving evidence. Reference lists of relevant articles and reviews were also hand-searched to identify additional sources.

### 2.2. Inclusion Approach and Evidence Prioritization

Given the interdisciplinary and mechanistically oriented scope of this review—spanning vascular biology, aging biology, CGM technology, nutrition, and pharmacology—both clinical (observational, randomized controlled trial) and preclinical (in vitro, animal model) studies were eligible for inclusion. Preclinical and animal studies were included deliberately and selectively, primarily where corresponding human outcome data are not yet available (e.g., lifespan data from the NIA Interventions Testing Program), and are explicitly identified as such throughout the text rather than presented as equivalent to human clinical evidence. Where multiple levels of evidence existed for a given claim (e.g., mechanistic, observational, and randomized trial data), the highest available level of evidence is presented, and lower tiers of evidence are used to provide mechanistic context, clearly labeled accordingly. Studies were prioritized for inclusion based on relevance to the review’s central themes, methodological quality, and, for clinical studies, sample size and study design. As a narrative rather than systematic review, formal risk-of-bias assessment and dual independent screening were not performed; selection and prioritization decisions were made by the author team through iterative discussion and consensus.

### 2.3. Use of Generative AI

Generative AI (GenAI: Claude Sonnet 4.5) was used solely to assist in generating graphical/illustrative elements presented in this manuscript. GenAI was not used for generating text, data, study design, data collection, analysis, or interpretation of results. All AI-assisted graphical content was reviewed by the authors for accuracy and appropriateness prior to inclusion.

## 3. Glycemic Variability, Vascular Biology, and Accelerated Aging

### 3.1. Postprandial Hyperglycemia and Endothelial Injury

The endothelium is the primary target of glycotoxic injury. Acute glucose spikes, even within the non-diabetic range (postprandial glucose 8–10 mmol/L), trigger rapid generation of reactive oxygen species via mitochondrial electron transport chain uncoupling, activate protein kinase C isoforms, and induce NF-κB-mediated inflammatory gene expression [7]. The resulting endothelial dysfunction—characterized by reduced nitric oxide bioavailability, increased adhesion molecule expression (ICAM-1, VCAM-1), and enhanced monocyte recruitment—is measurable within 60–90 min of a high-glycemic meal and is proportional to the magnitude of the glucose excursion [8].

Critically, glycemic variability (rapid oscillations between high and low glucose) appears more damaging to the endothelium than sustained mild hyperglycemia of equivalent mean value. In vitro studies demonstrate that intermittent high glucose activates apoptotic pathways in endothelial cells more potently than continuous high glucose exposure, supporting the concept that glucose swings—not merely averages—drive vascular injury [4].

Advanced glycation endproducts (AGEs), the non-enzymatic cross-linking products of glucose with proteins and lipids, accumulate progressively as a function of both mean glucose and exposure duration. AGEs stiffen collagen in arterial walls, increasing aortic pulse wave velocity and central pulse pressure, cross-link basement membrane components impairing microvascular permeability regulation, and activate RAGE (receptor for AGEs) to perpetuate inflammatory signaling [9]. These mechanisms directly link cumulative glycemic exposure to the arterial stiffness phenotype central to hypertension and heart failure with preserved ejection fraction (HFpEF).

Beyond its direct vascular and endothelial effects, glucose dysregulation also contributes to cardiometabolic risk through autonomic and sympathetic nervous system pathways. Chronic hyperglycemia has been linked to sympathetic overactivation and reduced parasympathetic tone, and mechanistic work has identified glucose-related signaling changes within cardiac sympathetic ganglia—including altered gap-junction-mediated glial signaling in the stellate ganglion—as a contributor to sympathetic overactivity and arrhythmic risk in type 2 diabetes [10]. While this pathway has been characterized primarily in established diabetes, it represents an additional, complementary route by which glycemic dysregulation may influence cardiovascular risk beyond direct vascular injury.

### 3.2. Glycemic Variability as a Cardiovascular Risk Factor Beyond HbA1c

Multiple epidemiological and mechanistic studies have established glycemic variability as an independent predictor of cardiovascular events beyond what HbA1c captures. Blood glucose oscillations are more strongly associated with atherosclerotic progression than steady-state hyperglycemia in type 2 diabetes cohorts, with postprandial glucose peaks independently predicting carotid intima-media thickness, coronary plaque instability, and major adverse cardiovascular events [11].

Among individuals hospitalized for acute coronary syndromes, wider glucose fluctuations—regardless of diabetic status—predicted recurrent MACE with an effect size comparable to conventional risk factors [12]. This observation is particularly important for the non-diabetic population encompassing both individuals with prediabetes and those with normal glucose tolerance who may experience clinically significant postprandial excursions that traditional screening methods fail to detect.

A systematic review of CGM use in non-diabetic individuals for cardiovascular prevention confirmed that glycemic variability metrics—specifically mean amplitude of glycemic excursions (MAGE), coefficient of variation, and time above range (TAR; >7.8 mmol/L)—correlate with subclinical atherosclerosis markers and inflammatory biomarkers in apparently healthy adults [13].

### 3.3. Glucose, Nutrient-Sensing Pathways, and the Biology of Aging

The mechanistic intersection of glucose metabolism and biological aging converges on four principal nutrient-sensing systems: (1) the insulin/IGF-1 signaling axis, (2) the mTORC1 (mechanistic target of rapamycin complex 1) pathway, (3) AMP-activated protein kinase (AMPK), and (4) NAD+-dependent sirtuins (SIRT1-7) [5]. Each of these pathways is acutely regulated by glucose and amino acid availability and collectively determines the balance between anabolic growth/storage and catabolic repair/maintenance programs.

Chronic postprandial hyperglycemia maintains mTORC1 in an activated state, suppressing autophagy—the cellular self-cleaning process that removes damaged organelles, misfolded proteins, and dysfunctional mitochondria. Autophagy impairment is a central feature of biological aging and age-related cardiovascular disease, as it underlies the accumulation of lipid-laden foam cells, dysfunctional vascular smooth muscle cells, and senescent endothelial cells in atherosclerotic plaques [14].

Conversely, periods of low glucose availability—as occur during intermittent fasting, caloric restriction, or AMPK-activating pharmacotherapy—reduce mTORC1 activity, activate autophagy, upregulate SIRT1 and SIRT3 (promoting mitochondrial biogenesis and oxidative stress resistance), and lower circulating IGF-1 levels. These changes collectively produce a biology analogous to the most robustly lifespan-extending interventions known in model organisms [6].

## 4. Continuous Glucose Monitoring: Technology, Metrics, and Use in Non-Diabetic Individuals

### 4.1. Technology Overview

Contemporary CGM systems measure glucose concentration in subcutaneous interstitial fluid via an amperometric electrochemical sensor positioned in the dermis. Readings are generated every 1–5 min, transmitted wirelessly to a receiver or smartphone, and displayed as real-time glucose traces with trend arrows. Current commercial devices (Abbott FreeStyle Libre 3, Dexcom G7, Medtronic Guardian 4) achieve mean absolute relative difference (MARD) values of 7–9% compared with laboratory-standard capillary glucose, adequate for lifestyle guidance applications [15].

Factory-calibrated devices have eliminated the requirement for fingerstick calibration, substantially reducing user burden. Sensor wear duration ranges from 10 to 15 days for current models, with experimental implantable biosensors demonstrating operational longevity of up to 6 months in vitro and computational projections suggesting >3-year implantable lifespans, which could enable continuous long-term metabolic phenotyping [16].

### 4.2. Key Metrics for Non-Diabetic Applications

For apparently healthy individuals using CGM as a preventive tool rather than a diagnostic instrument, the metrics presented in Table 1 are most clinically informative. It should be emphasized that, with the exception of TIR/TAR thresholds adapted from international consensus guidelines developed primarily for diabetic populations, the targets below are extrapolated, exploratory, or proposed by the authors as reference points for future research rather than validated clinical goals for cardiovascular prevention or longevity in non-diabetic individuals.

Proposed targets are intended as exploratory reference points for research and self-monitoring context, not as validated clinical thresholds; they require prospective validation in non-diabetic cohorts before adoption as care standards. It is important to emphasize that glycemic variability and postprandial glucose excursions within the non-diabetic range are common, physiological phenomena observed even in metabolically healthy individuals, and their presence does not, by itself, constitute evidence of disease or an indication for clinical intervention. The associations between glycemic variability metrics and cardiovascular risk discussed in this review are largely derived from observational and mechanistic studies and should be interpreted as hypothesis-generating signals warranting further research, not as diagnostic findings requiring correction in an apparently healthy individual.

### 4.3. Inter-Individual Variability in Postprandial Glucose Response

The landmark Weizmann Institute study by Zeevi and colleagues (2015) [3], conducted in 800 adults across the glycemic spectrum, provided definitive proof that postprandial glycemic responses to identical meals vary enormously between individuals. Factors explaining this variability—including gut microbiome composition, meal composition, body fat distribution, sleep quality, and physical activity preceding the meal—are highly personal and not predictable from standard nutritional advice. The study demonstrated that a machine-learning algorithm trained on personal CGM data, microbiome profiling, and lifestyle parameters could predict individual postprandial responses with high accuracy and generate personalized dietary recommendations that consistently reduced glycemic spikes [3].

This seminal finding has since been replicated in multiple cohorts and forms the scientific foundation for CGM-guided precision nutrition as a proposed preventive cardiology strategy. It highlights a limitation of population-average glycemic index tables: postprandial glucose response to a given food varies considerably between individuals, a distinction personal CGM data can help characterize—though individual variation in postprandial response is not in itself evidence of harm.

## 5. Fasting Protocols: Intermittent Fasting, Prolonged Fasting, and Longevity Mechanisms

### 5.1. Intermittent Fasting and Time-Restricted Eating

Intermittent fasting (IF) encompasses a range of protocols defined by alternating periods of eating and voluntary abstinence. The most widely studied variants include 16:8 time-restricted eating (TRE; eating within an 8 h window), 5:2 (two days per week of ~500 kcal intake), and alternate-day fasting (ADF; alternating ad libitum and ~25% calorie days) [19].

CGM data from subjects following TRE and IF protocols consistently demonstrate: extended periods of low, stable glucose during fasting windows; reduced postprandial glucose peaks and shorter duration of postprandial excursions; improved insulin sensitivity with each feeding cycle; and lower 24 h mean glucose and glycemic variability compared with conventional meal patterns [20]. An alternate-day fasting regimen in overweight non-diabetic adults over 8 weeks produced 8% body weight loss alongside significant reductions in fasting insulin, LDL cholesterol, and inflammatory markers [19]. These metabolic changes are directly visualizable on CGM traces, making CGM an ideal biofeedback tool for IF adherence and optimization.

From a cardiovascular prevention standpoint, IF produces improvements across multiple risk factor domains: blood pressure reduction (systolic BP −4 to −8 mmHg in short-term trials), LDL reduction, triglyceride reduction, and CRP reduction [21]. These effects are partly caloric but also attributable to the rhythmic activation of fasting-induced metabolic programs: hepatic ketogenesis, adipose lipolysis, and systemic downregulation of IGF-1 and insulin signaling.

### 5.2. Prolonged Fasting: Mechanisms of Cellular Repair and Proposed Cardiovascular Relevance

Prolonged fasting (PF), defined here as voluntary abstinence from caloric intake for 24–120 h with water and electrolyte maintenance, activates cellular programs not achievable by intermittent fasting protocols [22]. Much of the evidence supporting these cellular programs derives from preclinical models and short-term mechanistic studies in humans; direct evidence linking prolonged fasting to reduced cardiovascular events or mortality in non-diabetic adults is lacking, and the following mechanisms should be understood as biologically plausible rather than clinically established drivers of cardiovascular benefit. The key molecular events of PF are sequentially orchestrated by the progressive depletion of hepatic glycogen, the rise in ketone bodies (principally β-hydroxybutyrate), the fall in circulating insulin and IGF-1, and the activation of AMPK—each detectable as corresponding changes in CGM glucose dynamics.

- **Autophagy induction:** After approximately 18–24 h of fasting, autophagy flux has been shown to increase substantially in preclinical and limited human biomarker studies, peaking at 48–72 h. In experimental models, autophagy removes dysfunctional mitochondria (mitophagy), aggregated proteins, and lipid droplets from vascular cells, macrophages, and cardiomyocytes; these processes are mechanistically relevant to atherosclerotic plaque stability and cardiac aging, but direct evidence that fasting-induced autophagy improves clinical cardiovascular outcomes in humans is not yet available [23].
- **IGF-1 suppression:** Prolonged fasting has been shown to reduce circulating IGF-1 by approximately 40–60% within 3–5 days in human studies, a change hypothesized to suppress pro-growth, pro-senescence signaling [24]. IGF-1 reduction is associated with extended lifespan across model organisms from yeast to mammals; whether this translates into reduced cardiovascular risk or extended lifespan in humans practicing intermittent prolonged fasting has not been directly demonstrated.
- **mTORC1 inhibition:** In the absence of dietary amino acids and glucose, mTORC1 activity falls, derepressing autophagy and ULK1-mediated mitochondrial biogenesis in preclinical models. This mTORC1 suppression is mechanistically analogous to the pathway targeted by rapamycin, one of the most robustly lifespan-extending pharmacological interventions identified in model organisms; however, this analogy is mechanistic rather than evidence of equivalent clinical benefit, and rapamycin’s immunosuppressive effects and fasting’s physiological effects are not directly comparable in magnitude or human outcome data [14].
- **β-hydroxybutyrate as a longevity signal:** The principal ketone body produced during prolonged fasting, β-hydroxybutyrate (β-OHB), is proposed to function not merely as an energy substrate but as a signaling molecule, based largely on preclinical and in vitro evidence. In experimental models, it inhibits class I histone deacetylases (HDACs), increasing expression of FOXO3a transcription factor targets involved in oxidative stress resistance, and activates HCAR2 (GPR109A) to reduce NLRP3 inflammasome activity [24]. These anti-inflammatory mechanisms are plausibly relevant to coronary artery disease, but direct clinical evidence linking fasting-induced β-OHB elevation to cardiovascular outcomes in non-diabetic adults is not yet available [25].

CGM is uniquely positioned to guide safe prolonged fasting. The transition from glucose-fueled to ketone-fueled metabolism is marked by a characteristic CGM pattern: progressive decline in mean glucose toward 3.5–4.5 mmol/L with minimal variability, confirming metabolic switch completion and ketoadaptation. This pattern reflects successful metabolic adaptation to fasting, not evidence of downstream cardiovascular benefit.

### 5.3. Caloric Restriction: Evidence for Cardiovascular and Longevity Benefits

Caloric restriction (CR), defined as a reduction in caloric intake of 20–40% without malnutrition, is the most robustly replicated lifespan-extending intervention across model organisms [26]. The CALERIE (Comprehensive Assessment of Long-term Effects of Reducing Intake of Energy) trial—the first large randomized controlled trial of sustained CR in non-obese healthy humans—demonstrated that 25% CR over 2 years produced significant improvements in blood pressure, LDL cholesterol, CRP, and metabolic rate adaptation, alongside preservation of lean body mass [27].

From a glycemic perspective, CR produces lower fasting and postprandial glucose, reduced glycemic variability, and improved beta-cell function. CGM integration in CR studies allows real-time quantification of metabolic benefit accrual and supports adherence by providing immediate behavioral feedback. The CALERIE trial did not use CGM, but subsequent pilot studies have confirmed that CGM-guided CR achieves comparable metabolic improvements with higher participant engagement.

## 6. CGM-Guided Personalized Nutrition for Preventive Cardiology

### 6.1. The Case for Personalized Nutrition over Population-Average Guidelines

Standard dietary guidelines for cardiovascular prevention are derived from population-average metabolic responses to macronutrient categories and food groups. While valuable at the population level, these recommendations fail to account for the extraordinary inter-individual variability in glycemic responses that CGM has revealed [3]. A Mediterranean dietary pattern may produce ideal glycemic profiles in one individual while generating persistent postprandial excursions in another with a different microbiome composition or insulin kinetics phenotype.

Beyond its role in explaining inter-individual variability in postprandial glucose response, the gut microbiome itself represents a potential adjunctive target for glycemic risk reduction. A meta-analysis of clinical trials found that probiotic supplementation significantly reduced HbA1c and fasting blood glucose in individuals with type 2 diabetes compared with placebo [28], suggesting a possible role for microbiome-targeted approaches, such as probiotics, prebiotics, or synbiotics, as a low-risk adjunct to CGM-guided dietary personalization [28]. This evidence is derived primarily from individuals with established type 2 diabetes rather than apparently healthy, non-diabetic adults, and probiotic strain, dose, and duration varied considerably across included trials; whether microbiome-targeted supplementation meaningfully improves glycemic variability or cardiovascular risk in the non-diabetic population addressed in this review has not been established and represents a further avenue for future research within this framework.

CGM-guided personalized nutrition replaces this one-size-fits-all approach with an iterative, data-driven experimental paradigm: individuals test specific foods, meal compositions, meal timing sequences, and pre-meal physical activity bouts against their own CGM data, identifying personal dietary “responder” patterns that minimize glycemic variability and postprandial spikes. This approach has demonstrated superior glycemic outcomes compared with conventional dietary advice in multiple cohort studies and short-term randomized trials [29].

### 6.2. Practical CGM-Guided Dietary Strategies

The following dietary interventions have demonstrated consistent CGM-measurable glycemic benefit across population subgroups and are reinforced as personalized strategies through CGM biofeedback:

- Meal sequencing (protein and fiber before carbohydrates): Consuming dietary protein and non-starchy vegetables before starch reduces postprandial glucose peaks by 20–40% compared to identical meals consumed in carbohydrate-first order, mediated by GLP-1 release, gastric emptying delay, and pre-stimulation of the incretin axis [30].
- Post-meal physical activity: A 10–20 min walk within 30 min of a meal reduces CGM-measured postprandial glucose by 15–30% through insulin-independent GLUT4 translocation in skeletal muscle. CGM visualization of this effect dramatically enhances exercise motivation in healthy individuals [31].
- Carbohydrate quality and fiber density: Replacing refined carbohydrates with fiber-rich equivalents (legumes, intact whole grains) reduces MAGE by 25–35% through slowed digestion, short-chain fatty acid production, and GLP-1 stimulation.
- Circadian meal timing: Late evening meals (after 19:00) produce substantially higher and more prolonged postprandial glucose responses than identical morning meals due to circadian variation in insulin sensitivity (nadir at night). CGM data graphically display this temporal effect, promoting earlier dinner adoption [32].
- Dietary fat and protein composition: High-fat, moderate-protein meals blunt postprandial glucose peaks but may produce prolonged low-grade hyperglycemia 3–5 h later (particularly with saturated fat). CGM reveals these delayed responses invisible to standard 2 h postprandial glucose checks.

### 6.3. CGM as a Behavior Change Tool

Beyond its informational function, CGM exerts powerful behavioral influence through the mechanism of immediate salient feedback. In learning theory terms, CGM closes the feedback loop between food choice and metabolic consequence, converting a delayed, abstract consequence (cardiovascular risk accumulation over decades) into an immediate, visible, and personal response [33].

Studies pairing CGM with smartphone app-guided lifestyle coaching in individuals at high risk of type 2 diabetes report significant improvements in glycemic metrics, body weight, and dietary behavior adherence. Participants describe the experience as transformative: seeing a real-time glucose spike after a specific food item creates a visceral, associative learning response that reduces repeat consumption of that item more effectively than verbal education alone [34].

Proposed implementation framework for apparently healthy individuals: 2–4 weeks CGM “learning sprints” conducted once or twice annually, paired with a structured coaching curriculum (dietitian/lifestyle coach), focused on identifying personal dietary responders and non-responders, establishing individualized ΔPPG targets as coaching benchmarks, and documenting lifestyle modifications adopted based on CGM findings. This sprint-based model minimizes cost, avoids data fatigue, and concentrates behavioral learning in a high-engagement period [34].

## 7. Exercise, CGM, and Metabolic Health

### 7.1. Aerobic Exercise

During moderate aerobic exercise, contracting skeletal muscle increases glucose uptake up to 50-fold above resting rates through insulin-independent GLUT4 translocation, driving a characteristic CGM decline of 1–4 mmol/L depending on exercise intensity, duration, and pre-exercise glucose level [35]. This acute glucose-lowering effect is followed by a post-exercise period of enhanced insulin sensitivity lasting 24–48 h, during which subsequent meals produce smaller postprandial CGM excursions—a beneficial “carryover” effect detectable as a shift in the TAR profile across the following day.

Aerobic exercise training produces durable improvements in all CGM longevity metrics: lower 24 h mean glucose, increased TIR, reduced CV%, and lower MAGE. These effects are mediated by GLUT4 upregulation in skeletal muscle, improved hepatic insulin sensitivity, and mitochondrial biogenesis increasing oxidative capacity [36].

### 7.2. Resistance Training and High-Intensity Interval Training

Resistance exercise produces a distinct CGM pattern: minimal acute glucose decline during exercise (reliance on anaerobic glycolysis with transient catecholamine-mediated hepatic glucose output), followed by stable or slightly elevated early post-exercise glucose, and enhanced insulin sensitivity 12–24 h post-exercise. This profile makes resistance training particularly valuable for individuals prone to exercise-induced hypoglycemia and for preserving muscle mass—the principal site of postprandial glucose disposal—during aging [35].

High-intensity interval training (HIIT) characteristically produces a transient glucose spike (5–12 mmol/L) during sprint intervals due to catecholamine-driven hepatic glycogenolysis, followed by a rapid return to euglycemia and enhanced insulin sensitivity. CGM data in endurance-trained individuals show higher exercise-induced glucose spikes than untrained subjects—a paradoxical but physiologically expected finding reflecting superior hepatic counterregulatory response capacity [35]. Combined aerobic and resistance training produces the most favorable aggregate CGM profile and the most robust cardiovascular and longevity outcomes.

## 8. Low-Dose Metformin: AMPK Activation, Longevity, and Cardiovascular Prevention

### 8.1. Mechanisms of Action Beyond Glucose Lowering

Metformin (biguanide) is the most widely prescribed glucose-lowering medication globally, but its mechanisms extend far beyond hepatic gluconeogenesis suppression. At the molecular level, metformin primarily inhibits mitochondrial respiratory chain Complex I, reducing mitochondrial electron transport efficiency, lowering the ATP/AMP ratio, and thereby activating AMP-activated protein kinase (AMPK)—the master cellular energy sensor and the pharmacological analog of fasting or exercise [37].

AMPK activation by metformin produces a coordinated metabolic response s similar to caloric restriction: suppression of mTORC1 (inducing autophagy), activation of SIRT1 (enhancing mitochondrial biogenesis and oxidative stress resistance), phosphorylation of FOXO transcription factors (upregulating antioxidant and longevity genes), and inhibition of SREBP1-mediated lipogenesis [34]. At low doses (500–1000 mg/day), these intracellular effects occur with minimal systemic glucose lowering in euglycemic individuals, making the metabolic and longevity effects dissociable from hypoglycemia risk [38].

### 8.2. Longevity Evidence

Metformin is among the pharmacological agents with the strongest indirect signal for human longevity benefit, although this evidence derives from observational data in people with type 2 diabetes and preclinical models rather than dedicated trials in healthy non-diabetic adults. A landmark 2014 observational study by Bannister and colleagues, analyzing matched cohorts from UK primary care (*n* = 180,000), found that type 2 diabetic patients treated with metformin monotherapy had lower all-cause mortality than matched non-diabetic controls not taking metformin—a finding suggesting that metformin confers a survival advantage exceeding the benefit of simply not having diabetes [38]. This finding is observational and specific to individuals with type 2 diabetes; it does not directly demonstrate a survival benefit of metformin when used in apparently healthy, non-diabetic adults, a population in which no comparable mortality data currently exist.

Beyond this population-specificity concern, the Bannister finding—and similar observational associations between metformin use and survival—are subject to several well-recognized sources of bias that limit causal interpretation. The “healthy user effect” describes the tendency for individuals who reliably take a prescribed medication to also engage in other health-promoting behaviors (better dietary adherence, greater engagement with preventive care, higher socioeconomic status, better medication adherence generally) that independently predict better survival, potentially explaining much or all of an observed association without any causal drug effect. Indication bias is a related concern: metformin is preferentially prescribed as first-line therapy to younger, healthier, and more recently diagnosed individuals with type 2 diabetes, whereas alternative agents such as sulfonylureas are often reserved for patients with more advanced disease, longer diabetes duration, or contraindications to metformin (e.g., renal impairment)—meaning that metformin users and comparator groups may differ systematically on numerous unmeasured prognostic characteristics before treatment is even considered. The comparison in the Bannister study to non-diabetic controls is particularly susceptible to this problem, since diabetic and non-diabetic individuals differ in innumerable measured and unmeasured ways beyond metformin exposure itself. Immortal time bias may further inflate apparent benefit if metformin exposure or treatment duration was not handled with time-dependent methods, effectively crediting metformin with survival time that occurred before treatment was established or during periods when death was definitionally impossible for a person to remain classified as a “user.” Because these studies are observational and non-randomized, unmeasured confounding cannot be excluded by statistical adjustment alone, and the observed association may reflect the characteristics of the people who are prescribed and continue to take metformin rather than a causal effect of the drug itself.

Distinguishing these possibilities requires study designs specifically capable of addressing these biases: active-comparator, new-user cohort designs that compare metformin against another glucose-lowering agent initiated at a similar point in disease progression (rather than against non-users or non-diabetic controls) can reduce indication bias; explicit time-dependent exposure modeling can address immortal time bias; and Mendelian randomization approaches, which use genetic proxies for drug target modulation to approximate a randomized comparison, offer one non-randomized alternative. Ultimately, however, only a genuine randomized controlled trial can adequately exclude the healthy user effect and unmeasured confounding, which is precisely the role of the ongoing TAME (Targeting Aging with Metformin) trial. TAME randomizes non-diabetic older adults to metformin or placebo and is explicitly designed to test whether metformin causally reduces a composite of age-related morbidities; results are anticipated in approximately 2026–2027 [38]. The TAME (Targeting Aging with Metformin) trial—the first FDA-approved clinical trial explicitly testing a longevity intervention—is currently enrolling 3000 non-diabetic adults aged 65–79 to test whether metformin 1500 mg/day delays the composite incidence of age-related diseases (cardiovascular disease, cancer, dementia, and mortality) over 6 years [39]. TAME represents a paradigm shift in clinical medicine: treating aging itself as a therapeutic target. Interim biomarker data from TAME and predecessor studies suggests that metformin reduces circulating inflammatory markers (IL-6, TNF-α, CRP) and cellular senescence markers (p21, p16) in non-diabetic older adults.

Until TAME results are available, the claim that metformin extends lifespan or healthspan in non-diabetic individuals should be regarded as an unproven, biologically plausible hypothesis supported by observational associations that are themselves open to substantial alternative, non-causal explanations, rather than as an established or near-established clinical benefit.

In the Interventions Testing Program (ITP)—the most rigorous preclinical longevity screening program, using genetically diverse mouse populations across three independent sites—metformin extended median lifespan by 4–6% and maximum lifespan by 6% when initiated in mid-life [40]. Notably, the lifespan extension was larger in males, paralleling the sex-differential findings in human cardiovascular mortality data.

### 8.3. Cardiovascular Benefits in Non-Diabetic Populations

Beyond longevity, metformin demonstrates direct cardiovascular effects relevant to primary prevention. In the UK Prospective Diabetes Study (UKPDS) overweight subgroup, metformin reduced myocardial infarction risk by 39% compared with conventional therapy—an effect substantially larger than that attributable to its glucose-lowering action alone and suggesting direct vascular benefits [41]. This finding derives from a subgroup analysis of overweight patients with type 2 diabetes and has not been replicated in a dedicated trial of non-diabetic individuals; extrapolation to primary cardiovascular prevention in healthy adults is inferential.

Proposed cardiovascular mechanisms in non-diabetic individuals include: reduction in endothelial oxidative stress through Complex I inhibition and AMPK-mediated eNOS activation; anti-inflammatory effects via NF-κB suppression; anti-proliferative effects on vascular smooth muscle cells (relevant to atherosclerotic plaque stability); reduction in platelet aggregation; and modest LDL and triglyceride reduction [37].

In the context of CGM use, metformin’s primary cardiovascular-relevant CGM effect is a reduction in postprandial glucose excursions (ΔPPG reduced by approximately 0.5–1.0 mmol/L at 500–1000 mg/day in non-diabetic individuals) through hepatic glucose output suppression and modest enhancement of peripheral insulin sensitivity. CGM can objectively document this effect, providing mechanistic confirmation of drug activity and motivation for continued adherence.

### 8.4. Safety, Dosing, and Considerations in Apparently Healthy Individuals

Low-dose metformin (500 mg once or twice daily) is well tolerated in non-diabetic individuals with preserved renal function (eGFR > 45 mL/min/1.73 m^2^). The primary dose-limiting side effect—gastrointestinal intolerance (nausea, diarrhea)—is substantially mitigated by the extended-release formulation and gradual dose titration. Lactic acidosis, the historically feared complication, occurs at an incidence of approximately 3 per 100,000 patient-years and is essentially confined to contexts of renal failure, acute illness, or iodinated contrast exposure [37].

Vitamin B12 deficiency develops in approximately 10–15% of long-term metformin users through ileal calcium-dependent transport inhibition; annual B12 monitoring and supplementation if deficient is advisable. From a longevity perspective, B12 depletion is independently associated with elevated homocysteine and cognitive decline, making monitoring particularly important in this context.

Hypoglycemia with low-dose metformin monotherapy in non-diabetic individuals is exceedingly rare due to the drug’s insulin-independent mechanism; CGM during the initial titration period allows objective documentation of glycemic safety and reassurance.

## 9. Acarbose: Alpha-Glucosidase Inhibition, Postprandial Glycemic Control, and Lifespan Extension

### 9.1. Mechanism of Action

Acarbose is a pseudotetrasaccharide that competitively inhibits intestinal alpha-glucosidase and alpha-amylase enzymes in the brush border of the small intestinal epithelium, delaying the digestion and absorption of complex carbohydrates and disaccharides [42]. The clinical consequence is a blunting of postprandial glucose peaks: in non-diabetic and prediabetic individuals, acarbose at doses of 50–100 mg three times daily with meals reduces postprandial glucose excursion by 1.5–2.5 mmol/L and AIx (augmentation index) by 4–8%—directly addressing the glucose spike-driven vascular injury mechanism.

Acarbose also modifies the gut microbiome: by delivering undigested oligosaccharides to the colon, it promotes growth of Bifidobacterium and Lactobacillus species and increases colonic production of short-chain fatty acids (SCFAs), particularly propionate and butyrate. SCFAs activate GLP-1 secretion from L-cells, enhance intestinal barrier function, and exert systemic anti-inflammatory effects—adding a microbiome-mediated cardiovascular benefit layer to direct glycemic effects [43].

### 9.2. Longevity Evidence from the Interventions Testing Program

Acarbose is one of only a handful of compounds to achieve robust longevity extension in the rigorous NIA Interventions Testing Program. Harrison and colleagues demonstrated that acarbose fed to genetically diverse mice throughout life extended median lifespan by 22% in males and 5% in females [44]. This sex-differential effect—larger in males—is mechanistically attributed to greater postprandial glucose and IGF-1 reduction in male mice, as females maintain lower basal glucose levels with less acarbose-responsive excursion.

The longevity mechanism is primarily attributed to IGF-1 reduction (circulating IGF-1 fell by approximately 30% in acarbose-treated male mice), consistent with the extensive literature linking IGF-1 suppression to lifespan extension across species [45]. Secondary contributors include reduced oxidative stress (lower 8-isoprostane), preserved insulin sensitivity throughout aging, and the anti-inflammatory effects of enhanced SCFA production. These findings are derived from murine models; direct translation of lifespan effects to humans has not been established.

### 9.3. Cardiovascular Evidence in Human Studies

The cardiovascular evidence for acarbose, summarized below, is drawn entirely from individuals with impaired glucose tolerance or type 2 diabetes; no trial has yet evaluated cardiovascular outcomes of acarbose in a normoglycemic, apparently healthy population.

The STOP-NIDDM trial (n = 1429, individuals with impaired glucose tolerance) demonstrated that acarbose 100 mg three times daily reduced the incidence of type 2 diabetes by 25% over 3.3 years [46]. In a prespecified but secondary analysis of this trial—not the primary endpoint, and conducted in individuals with impaired glucose tolerance rather than normoglycemic adults—acarbose was associated with a reduced risk of any cardiovascular event (HR 0.51; 95% CI 0.28–0.95) and myocardial infarction (HR 0.09; 95% CI 0.01–0.72) [46]. These estimates were based on only 47 total cardiovascular events across the trial, far fewer than the several hundred to nearly one thousand events typical of adequately powered cardiovascular outcomes trials; this small numerator produces the extremely wide confidence intervals observed, particularly for myocardial infarction, and indicates substantial statistical imprecision. Critically, this finding has not been replicated: the Acarbose Cardiovascular Evaluation (ACE) trial, a purpose-built cardiovascular outcomes trial in 6526 patients with coronary heart disease and impaired glucose tolerance—more than 100 times the event count of STOP-NIDDM—found no reduction in major adverse cardiovascular events with acarbose (HR 0.98; 95% CI 0.86–1.11) [47]. The most likely explanation for the discrepancy, as noted by the ACE investigators themselves, is that the original STOP-NIDDM cardiovascular finding represented a chance result in an underpowered study not designed to formally evaluate cardiovascular outcomes [48].

While the STOP-NIDDM cardiovascular results have been subject to methodological discussion (the trial was not powered for cardiovascular endpoints), the biological plausibility is strong: the observed reductions in post-meal glucose, blood pressure, and CRP are consistent with established mechanisms of cardiovascular risk reduction. A meta-analysis of 7 trials in type 2 diabetes (n = 2280) confirmed that acarbose reduces the risk of myocardial infarction by 64% and any cardiovascular event by 35% compared with placebo, with effects attributable to postprandial glucose reduction [49]. This meta-analysis was conducted exclusively in individuals with type 2 diabetes; no dedicated cardiovascular outcomes trial of acarbose in non-diabetic, apparently healthy adults has been performed.

### 9.4. CGM Integration with Acarbose Therapy

The synergy between acarbose and CGM is particularly direct: acarbose specifically targets postprandial glucose spikes—the very metric most visibly and immediately represented in CGM data. CGM allows personalization of acarbose dosing (adjusting dose based on meal carbohydrate content, guided by prior CGM spike data for that meal category), titration to the minimum effective dose reducing postprandial glucose rises to individual target thresholds (e.g., ΔPPG < 1.5 mmol/L), and documentation of the temporal and magnitude-specific glycemic response to each meal, confirming drug activity and identifying meal categories where higher or lower doses are appropriate.

Gastrointestinal side effects (flatulence, bloating) from acarbose—caused by colonic fermentation of undigested carbohydrates—are the principal barrier to adherence and can be substantially mitigated by low initial dosing (25 mg with one meal per day, titrating over 4–6 weeks) and dietary adjustment toward lower-carbohydrate meals identified through CGM as high-spike-risk foods.

**Figure 1:**
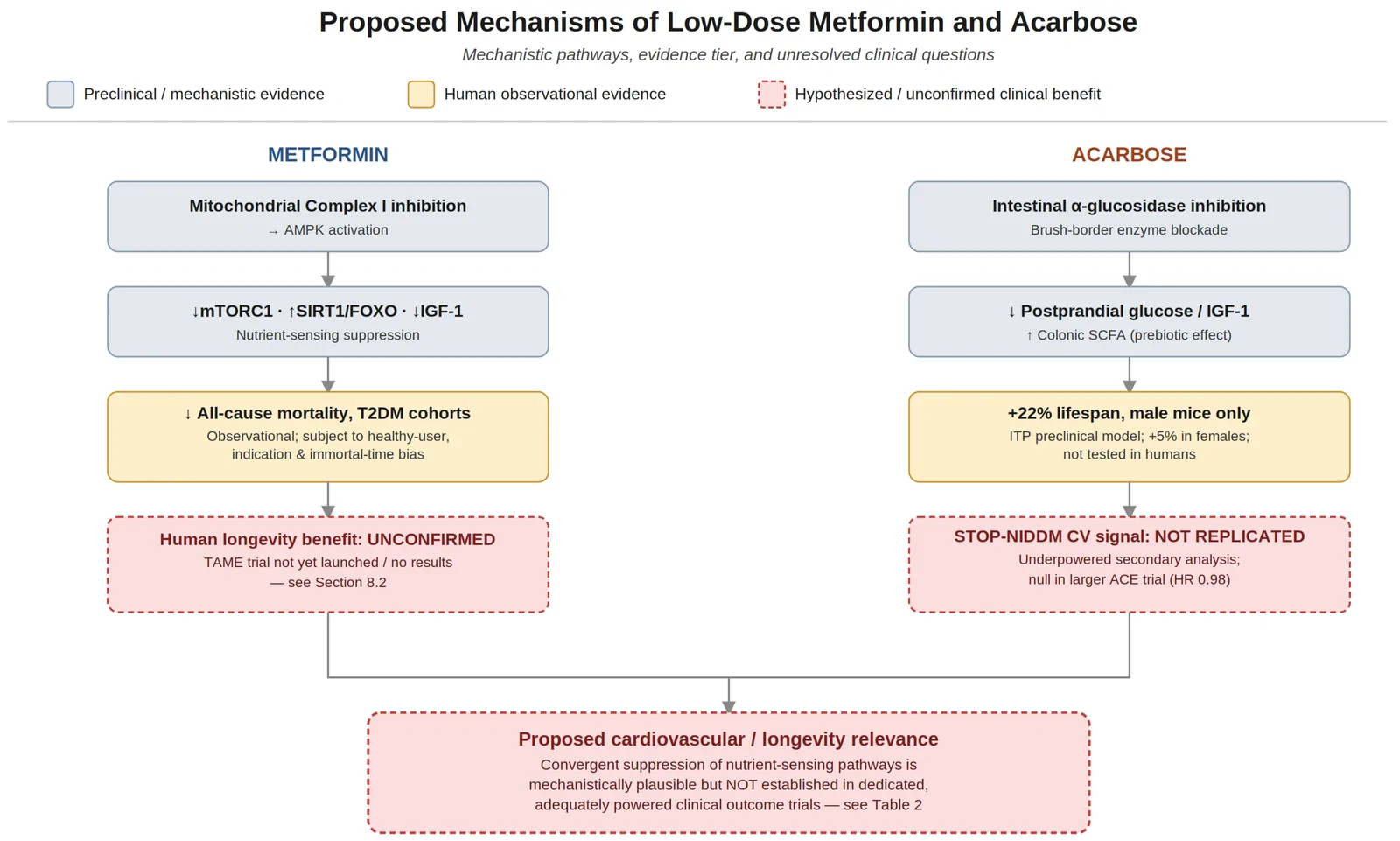
Proposed mechanisms of low-dose metformin and acarbose, by evidence tier. Metformin (left) inhibits mitochondrial Complex I, activating AMPK and suppressing mTORC1/IGF-1 signaling; this mechanistic pathway (gray) is well established in preclinical and cell-based studies [3,35]. Its association with reduced mortality in observational cohorts of individuals with type 2 diabetes (amber) [36] is subject to healthy-user, indication, and immortal-time bias (Section 8.2), and a human longevity benefit in non-diabetic adults remains unconfirmed pending the (currently unlaunched) TAME trial (red, dashed) [38]. Acarbose (right) inhibits intestinal α-glucosidase, reducing postprandial glucose and IGF-1 while promoting colonic short-chain fatty acid production (gray) [40]. Preclinical lifespan extension was observed in male but not female mice in the NIA Interventions Testing Program (amber) [42], and an early cardiovascular benefit signal from a secondary, underpowered STOP-NIDDM analysis (red, dashed) [46] was not replicated in the larger, purpose-built ACE cardiovascular outcomes trial [47]. The convergence of both agents on nutrient-sensing pathway suppression is mechanistically plausible but has not been established as a cause of cardiovascular or longevity benefits in adequately powered human trials. Abbreviations: AMPK, AMP-activated protein kinase; CHO, carbohydrate; ER, extended-release; FOXO, forkhead box O; GLP-1, glucagon-like peptide-1; IGF-1, insulin-like growth factor-1; ITP, Interventions Testing Program (NIA); LDL, low-density lipoprotein; MI, myocardial infarction; mTORC1, mechanistic target of rapamycin complex 1; NF-κB, nuclear factor kappa B; RCT, randomised controlled trial; SCFA, short-chain fatty acid; SIRT1, sirtuin-1; T2DM, type 2 diabetes mellitus; TG, triglyceride; Dosing recommendations are for apparently healthy non-diabetic adults with preserved renal function (eGFR > 45 mL/min/1.73 m^2^).

Figure 1 illustrates the primary molecular mechanisms of low-dose metformin and acarbose and their convergence on shared longevity-relevant pathways. Metformin acts through mitochondrial Complex I inhibition and downstream AMPK activation, producing a caloric restriction-mimetic intracellular environment, while acarbose operates through a complementary intestinal mechanism, combining direct postprandial glucose attenuation with prebiotic-mediated anti-inflammatory effects. Despite their mechanistically distinct primary targets, both agents converge on the suppression of overactive nutrient-sensing pathways—reducing IGF-1, mTORC1 activity, and chronic inflammation—the principal molecular drivers of accelerated cardiovascular aging reviewed in Section 3. CGM integration provides the real-time titration and efficacy-monitoring layer that allows both interventions to be personalised and objectively confirmed at the individual level. This framework is proposed as a structured hypothesis for clinical investigation and should not be interpreted as a validated treatment protocol; its individual components require prospective validation, ideally with hard cardiovascular endpoints, before broad clinical adoption.

## 10. Integrated Framework: A Research Model for CGM-Guided Metabolic Optimization in Apparently Healthy Individuals

### 10.1. Synergistic Mechanisms

CGM, structured fasting, low-dose metformin, and acarbose operate through complementary and partially non-overlapping mechanisms that together address the principal molecular hallmarks of cardiovascular aging: chronic postprandial glycemic burden, nutrient-sensing pathway overactivation, autophagy deficiency, chronic inflammation, endothelial oxidative stress, and vascular stiffening.

Their synergies are mechanistically coherent: acarbose blunts the initial postprandial glucose spike that metformin does not primarily target (metformin acts on fasting/hepatic glucose output); metformin activates AMPK during interprandial periods as fasting does between meals; prolonged fasting activates autophagy at a depth neither metformin nor acarbose alone can achieve; and CGM provides the real-time metabolic transparency that allows all three interventions to be personalized, titrated, and confirmed to be working.

### 10.2. A Four-Phase Research Framework: Proposed Model for Prospective Validation

We propose the following evidence-informed implementation framework for preventive CGM-guided longevity medicine in apparently healthy adults (age ≥ 35, no diabetes, no major organ impairment).

The age threshold of 35 years is a pragmatic starting point reflecting the age at which cardiometabolic risk factors commonly begin to manifest and at which preventive intervention may plausibly influence long-term cardiovascular trajectory, rather than an evidence-based cutoff derived from dedicated studies of this framework. We note that this threshold falls below the validated age range of the SCORE2 risk algorithm (40–69 years) discussed below; individuals aged 35–39 interested in this framework may still benefit from qualitative risk factor assessment and family history review even though formal SCORE2 calculation is not validated in this age range.

#### 10.2.1. Phase 1: Metabolic Phenotyping (Weeks 1–4)

Baseline metabolic assessment: fasting glucose, HbA1c, fasting insulin, HOMA-IR, fasting lipids, hsCRP, renal function (eGFR, creatinine), B12, vitamin D.

CGM sprint 1 (14 days): Standard diet, documenting baseline postprandial responses to habitual food categories, meal timing patterns, and exercise responses.

Output: Personal “glycemic fingerprint”—top 5 high-spike meals, personal optimal meal timing window, postprandial activity response magnitude.

#### 10.2.2. Phase 2: Dietary Optimization (Weeks 5–12)

CGM sprint 2 (14 days): Testing dietary modifications—meal sequencing, food substitutions, post-meal walks—against CGM feedback. Confirming personalized dietary strategy.

Target metrics: TIR > 97%, TAR < 3%, CV% < 20%, ΔPPG < 1.5 mmol/L for test meals.

Coaching: Registered dietitian or certified health coach review of CGM data with participant.

#### 10.2.3. Phase 3: Fasting Protocol Integration (Months 3–6)

Introduce TRE 16:8 initially, using CGM to confirm fasting glucose decline and stable euglycemia during fasting window.

Progress to monthly 24–48 h prolonged fasting cycles for individuals tolerating TRE well, with CGM confirming metabolic switch (stable low glucose 3.5–4.5 mmol/L, no symptomatic hypoglycemia).

Monitor for muscle mass preservation: DEXA or bioimpedance every 6 months.

Prolonged fasting protocols require careful patient selection and monitoring; contraindications (e.g., history of eating disorders, frailty, pregnancy, insulin-treated diabetes) and potential adverse psychological or physiological effects have not been systematically studied in apparently healthy populations pursuing fasting for longevity purposes rather than for an established medical indication.

#### 10.2.4. Phase 4: Pharmacological Augmentation (If Indicated; From Month 6)

Candidate criteria for low-dose metformin: HOMA-IR > 2.0, persistent TAR > 5% after dietary optimization, family history of type 2 diabetes or premature CVD, interest in longevity pharmacology after informed discussion.

Metformin initiation: 500 mg extended-release with dinner, titrate to 500 mg twice daily over 4 weeks. Annual B12 monitoring.

Candidate criteria for acarbose: persistent high-spike meals (ΔPPG > 2.0 mmol/L) despite dietary optimization, preference for carbohydrate-rich diet, or combined CGM evidence of postprandial risk.

Acarbose initiation: 25 mg with largest meal, titrate over 6 weeks to 50–100 mg three times daily. CGM confirmation of dose-dependent spike reduction.

It is plausible that lower acarbose doses than those used in STOP-NIDDM—for example, 25–50 mg with meals—could achieve meaningful attenuation of postprandial glucose excursions in non-diabetic individuals while substantially reducing the gastrointestinal side effect burden that limits adherence (Section 11.4), given that gastrointestinal tolerability is dose-dependent; however, this has not been formally tested in a dedicated dose-ranging study in this population. We propose that CGM data could, in principle, support an individualized “minimum effective dose” approach: rather than titrating to a fixed target dose, dose could be titrated upward only as needed to achieve a pre-specified reduction in CGM-measured postprandial glucose excursion (e.g., ΔPPG), stopping at the lowest dose that achieves this target for a given individual. This approach could optimize the risk–benefit ratio by minimizing gastrointestinal burden while maintaining glycemic benefit, but it is a proposed research concept rather than a validated clinical strategy, and would require prospective testing before adoption.

These doses are presented as illustrative starting points for clinical consideration and future research, not as definitive, evidence-based dosing recommendations for apparently healthy, non-diabetic adults. The metformin dosing schedule is extrapolated from diabetes management guidelines; no dose-ranging study has established an optimal dose for cardiovascular prevention or longevity purposes in non-diabetic individuals, nor is it established that the minimum effective dose for non-glycemic benefit is the same as that used for glycemic control in diabetes. The acarbose dosing schedule reflects doses used in STOP-NIDDM, a trial conducted in individuals with impaired glucose tolerance rather than normoglycemic adults; the substantially higher per-kilogram doses used in preclinical lifespan studies (e.g., the NIA Interventions Testing Program, approximately 1000 mg/kg of diet in mice) are not directly translatable to human dosing and are noted here only for mechanistic context. Dedicated dose-ranging studies of either agent in apparently healthy, non-diabetic adults are lacking, and this represents an important gap for future research.

Combination therapy: Metformin and acarbose have complementary, non-overlapping primary mechanisms and no known pharmacokinetic interaction. However, combined use has not been directly studied in apparently healthy, non-diabetic populations, and the two agents’ independent gastrointestinal side effect profiles may be additive rather than offsetting. Combination therapy should therefore not be assumed to be broadly appropriate; it should be considered only after individualized clinical evaluation and risk assessment, with gradual, staggered initiation of each agent under medical supervision to help distinguish the source of any adverse effects (see Section 11). Pharmacological augmentation, as described in this framework, is intended exclusively for physician-supervised use following individualized medical evaluation; it is not intended for self-initiated or unsupervised use (see Section 11.5).

It should be emphasized that metformin and acarbose are not currently approved for use in non-diabetic, apparently healthy individuals for cardiovascular prevention or longevity purposes. Their inclusion in this framework is conceptual, reflecting mechanistic rationale and signals from observational and small trial data; off-label prescription in this population should occur only within a research protocol or under specialist supervision after individualized risk–benefit assessment, and is not a general recommendation.

#### 10.2.5. Annual Monitoring

Annual CGM sprint (14 days) to assess metabolic phenotype evolution and treatment response.

Biannual metabolic panel, B12, renal function.

Longitudinal tracking of CGM longevity metrics: trend in mean glucose, CV%, TIR over successive annual sprints.

**Figure 2:**
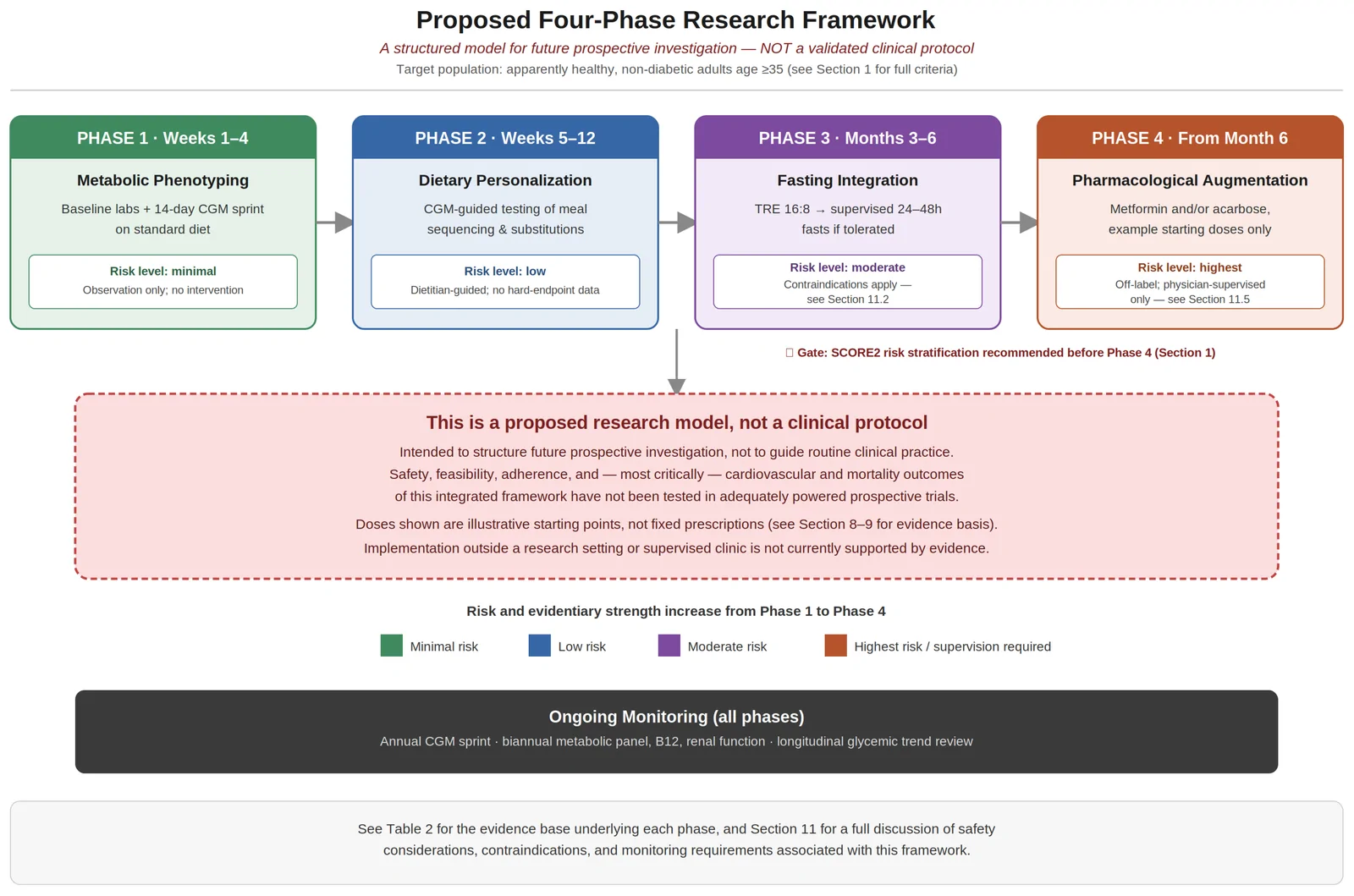
Proposed four-phase research framework for CGM-guided metabolic optimization in apparently healthy, non-diabetic adults. This framework is presented as a structured model intended to guide future prospective investigation, not as a validated clinical protocol for routine implementation (Section 10.2). Risk and evidentiary uncertainty increase across phases, from minimal-risk observational CGM phenotyping (Phase 1, green) through dietary personalization (Phase 2, blue) and supervised fasting integration (Phase 3, purple, subject to contraindications detailed in Section 11.2) to pharmacological augmentation (Phase 4, orange), which involves off-label use of metformin and/or acarbose and is intended exclusively for physician-supervised use following formal cardiovascular risk stratification (e.g., SCORE2) and individualized risk–benefit assessment (Section 11.5). Doses shown in Phase 4 are illustrative starting points for clinical consideration, not fixed prescriptions (Section 8 and Section 9). Safety, feasibility, adherence, and cardiovascular or mortality outcomes of this integrated framework have not been established in prospective trials; see Table 2 for the evidence base underlying each phase and Section 11 for a complete discussion of safety considerations.

Figure 2 presents the proposed four-phase research framework for CGM-guided metabolic optimization in apparently healthy adults, translating the mechanistic evidence reviewed in Section 3, Section 4, Section 5, Section 6, Section 7, Section 8 and Section 9 into a structured, stepwise model intended to guide future prospective investigation, not a validated clinical protocol. Each phase builds upon the metabolic information generated by the preceding one, with risk and evidentiary uncertainty increasing across phases; formal cardiovascular risk stratification is recommended before consideration of Phase 4 (pharmacological augmentation), which is intended exclusively for physician-supervised use (Section 11.5).” This framework is proposed as a structured research model intended to guide future prospective investigation, not as a validated clinical protocol for routine implementation. Its application outside a research setting or dedicated preventive cardiology/longevity clinic with appropriate medical supervision is not currently supported by available evidence.

Table 2 summarizes, for each major intervention discussed in this review, the population studied, study design, endpoints assessed, strength of evidence, key limitations, and applicability to apparently healthy, non-diabetic adults, to help distinguish established clinical evidence from mechanistic or extrapolated evidence. Taken together, safety, feasibility, long-term adherence, psychological impact, and—most critically—cardiovascular and mortality outcomes of this integrated framework have not been tested in adequately powered prospective studies. We present it here as a testable research model rather than a recommendation for clinical practice.

## 11. Safety Considerations

The interventions discussed in this review are not without risk, and their combination in apparently healthy individuals—outside the context of an established medical indication—warrants explicit caution. This section consolidates safety considerations relevant to each component of the proposed framework.

### 11.1. CGM Use: Psychological, Behavioral, Dermatological, and Financial Considerations

Continuous glucose monitoring, by making previously invisible physiological fluctuations constantly visible, carries a risk of overinterpretation. Emerging evidence on CGM as a behavior change tool highlights that, alongside its benefits, sustained self-monitoring of glucose data can carry psychological burdens that remain incompletely characterized, particularly in users without diabetes [50]. Normal, non-pathological glucose variability can be misperceived as a health threat, potentially fostering anxiety, hypervigilance around food choices, and orthorexic eating patterns—an excessive preoccupation with “correct” eating that can itself become clinically significant [51]. This risk may be particularly relevant in individuals with a personal or family history of disordered eating, for whom CGM-guided dietary restriction is not recommended without specialist psychological input. Users and clinicians should be aware that day-to-day and meal-to-meal glucose variability within the normal range is physiological, not necessarily pathological, and that the “longevity-optimal” targets discussed in this review (Table 1) are exploratory reference points rather than thresholds requiring corrective action.

Evidence specifically addressing the psychological effects of CGM in non-diabetic populations remains limited. A recent systematic review of CGM use for guiding lifestyle interventions in non-diabetic adults found that most available studies were small, short-term, and often lacked a controlled comparison group, limiting confidence in both the behavioral efficacy and the psychological safety of this approach [52]. Rigorous, validated-instrument quality-of-life data—using standardized anxiety, disordered-eating, or quality-of-life questionnaires—specifically in non-diabetic, CGM-guided preventive contexts are, to our knowledge, not yet available; most quality-of-life CGM research to date has been conducted in individuals with established diabetes, for whom the psychological context (disease management rather than optimization in an already-healthy individual) differs substantially and may not generalize.

A related and underexamined question is whether CGM-driven behavior change is durable once the sensor is removed. Real-world data from a consumer CGM-integrated nutrition app found that daily food-logging engagement fell from approximately 63% during active sensor wear to approximately 2% after sensor removal, illustrating how strongly behavioral engagement may depend on the continued presence of real-time biological feedback rather than reflecting a lasting internalized change [53]. Other studies, primarily in diabetic or prediabetic populations, have reported dietary improvements persisting for up to 13 months after CGM discontinuation [54], suggesting durability is not uniformly poor but may depend heavily on the structure, duration, and support accompanying CGM use. The “sprint” model proposed in this framework—repeated 2–4 week periods of CGM use, once or twice annually—is intended to balance behavioral reinforcement against cost and monitoring burden, but this specific intermittent-use pattern has not itself been validated for producing durable behavior change, and should be understood as a proposed, testable design choice rather than an established best practice.

Given these considerations, we propose the following concrete mitigation strategies, intended to reduce psychological risk while preserving the behavioral benefits of CGM feedback. First, individuals should be screened for a personal or family history of eating disorders, clinically significant anxiety, or perfectionistic or orthorexic tendencies prior to initiating CGM-guided dietary change; a positive screen should prompt either exclusion from CGM-guided dietary intervention or its use only with concurrent, structured psychological support. Second, any coaching accompanying CGM use should be delivered by an appropriately credentialed professional—for example, a registered dietitian or certified diabetes care and education specialist with specific training in CGM interpretation—and should emphasize flexible, sustainable dietary change over rigid rules or elimination-based restriction, with explicit guidance on interpreting CGM data without over-reacting to individual glucose excursions. Third, coaching and any accompanying educational materials should explicitly frame day-to-day and meal-to-meal glucose variability within the non-diabetic range as a normal physiological phenomenon rather than a personal failure or a marker of disease, consistent with the interpretation of Table 1 discussed above and in Section 11.6. Fourth, given the absence of validated quality-of-life instruments specific to this context, clinicians and researchers implementing CGM-guided frameworks in non-diabetic individuals should, at minimum, informally monitor for emerging signs of anxiety, disordered eating, or reduced quality of life—such as loss of dietary spontaneity or reduced enjoyment of social eating—over the course of CGM use, and should discontinue CGM-guided intervention if these emerge.

We do not consider the current evidence sufficient to conclude that CGM-guided behavior change in non-diabetic individuals is either durably effective or psychologically safe at scale; both questions represent priorities for future research, ideally through adequately powered randomized trials incorporating validated psychological and quality-of-life instruments, structured coaching protocols with defined qualifications, and follow-up extending well beyond the period of active sensor use to assess durability of behavior change.

Adhesive-related skin reactions are common with CGM use. Allergic and irritant contact dermatitis, most frequently attributed to acrylate-based adhesives (including isobornyl acrylate and colophony), have been reported with increasing frequency as CGM use has expanded, with some series reporting skin irritation or reaction in up to 90% of long-term device wearers and confirmed allergic sensitization in a substantial subset on patch testing [55]. Rotating sensor sites, barrier films, and alternative adhesive products may reduce but not eliminate this risk; individuals with known adhesive or acrylate allergy should be counseled accordingly before initiating CGM use.

Cost represents a further practical barrier, particularly relevant to this review’s target population of apparently healthy, non-diabetic adults, for whom CGM is rarely covered by insurance. Out-of-pocket costs for non-diabetic CGM use range from approximately USD 80 to 300 per month depending on device and subscription model [56] a recurring expense that may limit equitable access to CGM-guided approaches and should be weighed against their currently unproven clinical benefit in this population [56].

### 11.2. Fasting: Contraindications and Precautions

Intermittent and prolonged fasting protocols are contraindicated or require specialist supervision in several populations, including individuals with a current or historical eating disorder, pregnancy and lactation, frailty or underweight status, insulin-treated diabetes (risk of hypoglycemia), significant hepatic or renal impairment, and those on medications requiring consistent food intake. Fasting has been identified as a risk factor for the development of disordered eating and orthorexia nervosa in observational studies, and clinicians are advised to screen for these risks before recommending fasting-based interventions [51,57]. Prolonged fasting in particular carries risks of electrolyte disturbance and hypotension, and, on refeeding, of refeeding syndrome—a potentially serious shift in fluids and electrolytes triggered by the reintroduction of food after a prolonged period of caloric restriction [58]. Psychological effects of prolonged fasting have not been systematically studied in individuals pursuing it for longevity rather than an established medical indication, and adherence and safety data in this context remain limited.

Prolonged fasting is also associated with a reproducible pattern of endocrine and hemodynamic adaptations that carry clinical risk if unmonitored, including transient hypogonadism, relative hypothyroidism, hypercortisolemia, catabolic loss of lean mass, and orthostatic intolerance [59]. Electrolyte disturbances, particularly hyponatremia and hypokalemia, may develop during prolonged fasting due to reduced intake combined with ongoing renal and gastrointestinal losses, and can predispose to cardiac arrhythmia if uncorrected; maintaining adequate sodium and potassium intake during supervised fasting protocols, and periodic electrolyte monitoring for fasts extending beyond 24–48 h, are recommended precautions. Orthostatic hypotension, manifesting as dizziness or lightheadedness on standing, is common during fasting due to reduced circulating volume and should prompt gradual postural changes and adequate fluid and sodium intake; persistent or severe symptoms warrant fast discontinuation and medical evaluation.

### 11.3. Metformin: Renal Function, Lactic Acidosis, GI Tolerability, B12 Monitoring, and Exercise Considerations

Low-dose metformin should be avoided or dose-adjusted in individuals with reduced renal function; current consensus guidance recommends against initiating metformin at an eGFR between 30 and 44 mL/min/1.73 m^2^ and contraindicates its use below 30 mL/min/1.73 m^2^ due to the risk of drug accumulation and lactic acidosis [60]. Metformin-associated lactic acidosis (MALA) is a rare but serious complication, with estimated incidence ranging from approximately 3 to 47 cases per 100,000 patient-years across observational studies, and a case fatality rate historically estimated at 25–50% when it occurs [61,62]. The risk of MALA is concentrated overwhelmingly in individuals with significant renal impairment, hypoxia, sepsis, or acute illness, and is exceedingly rare in individuals with preserved renal function; nonetheless, given the population addressed in this review is by definition apparently healthy, routine renal function monitoring (see below) and temporary discontinuation during acute illness, dehydration, or perioperative periods are prudent precautions rather than theoretical concerns.

The effects of metformin on exercise performance and lactate metabolism in physically active individuals warrant specific mention, given that structured physical activity is a common component of preventive cardiology and longevity-oriented interventions. Metformin has been shown to modestly blunt some favorable adaptations to aerobic exercise training in certain contexts, and, due to its mechanism of action, can transiently increase circulating lactate during intense exercise; while this is not generally considered clinically dangerous in individuals with normal renal and hepatic function, it may cause perceptible symptoms (e.g., early fatigue) during high-intensity exercise and should be discussed with physically active patients considering metformin use.

Metformin should also be temporarily discontinued during acute illness, dehydration, or before iodinated contrast administration. Gastrointestinal intolerance is common and dose-limiting; extended-release formulations and gradual titration mitigate this. Long-term use is associated with vitamin B12 deficiency, demonstrated in a randomized placebo-controlled trial to cause a mean 19% reduction in serum B12 concentration over 4.3 years of treatment, with a number needed to harm of approximately 14 [63]. Periodic monitoring and supplementation if deficient is therefore advisable, particularly given the additional relevance of B12 status to cardiovascular and cognitive health in this population. Elderly individuals and those on polypharmacy warrant particular caution, given age-related declines in renal function, higher baseline prevalence of B12 insufficiency, and increased potential for drug interactions; metformin dosing and monitoring intervals should be adjusted accordingly in these subgroups.

A reasonable monitoring schedule includes renal function (eGFR) assessment prior to initiation, at 3 months, and at least annually thereafter (more frequently if eGFR is reduced or declining), and serum vitamin B12 assessment at baseline and annually, or sooner if symptoms of deficiency (fatigue, paresthesia, cognitive changes) develop.

### 11.4. Acarbose: Gastrointestinal Tolerability, Hypoglycemia Management, and Contraindications

Gastrointestinal adverse effects are common with acarbose and represent the principal barrier to adherence. In placebo-controlled trials, flatulence occurred in approximately 74% of acarbose-treated patients (versus 29% with placebo), diarrhea in approximately 31% (versus 12%), and abdominal pain in approximately 19% (versus 9%) [64]. These effects are generally most pronounced in the first weeks of treatment and diminish over time with continued use and gradual dose titration; nonetheless, gastrointestinal intolerance remains the most common reason for treatment discontinuation, with discontinuation rates due to adverse effects reported as high as 30–37% in some studies [65]. Acarbose is contraindicated in individuals with inflammatory bowel disease, colonic ulceration, partial intestinal obstruction, or a predisposition to intestinal obstruction, given rare postmarketing reports of ileus and pneumatosis cystoides intestinalis [64]. Although acarbose does not cause hypoglycemia when used alone, its combination with other glucose-lowering agents (sulfonylureas, insulin, or, in the context of this review, structured fasting protocols) increases hypoglycemia risk. Critically, if hypoglycemia occurs during acarbose therapy, it must be treated with oral glucose (dextrose) rather than sucrose (table sugar) or complex carbohydrates, because acarbose inhibits the intestinal breakdown of sucrose and starch, delaying and blunting the glycemic response needed for rapid correction; severe hypoglycemia may require intravenous glucose or glucagon. This distinction is clinically important and should be explicitly communicated to any individual using acarbose, particularly if combined with fasting protocols within the proposed framework.

### 11.5. Combined Pharmacological Use and Medical Supervision

The framework proposed in this review includes off-label use of metformin and acarbose in individuals without an approved indication for either drug. This should not be undertaken without individualized medical evaluation, including assessment of renal and hepatic function, contraindications, and interacting medications, and ongoing supervision. Self-initiated or unsupervised use of these agents for longevity or cardiovascular prevention purposes is not supported by current evidence and is not recommended by the authors.

### 11.6. Extrapolation of Diabetes-Derived Thresholds

As discussed in relation to Table 1, the CGM metric thresholds referenced throughout this review are largely extrapolated or adapted from consensus guidelines developed for diabetic and prediabetic populations [17]. Applying these thresholds to apparently healthy individuals without validation carries a risk of pathologizing normal physiology, prompting unnecessary anxiety or intervention, and diverting attention from established cardiovascular risk factors with stronger evidence bases.

### 11.7. Risk–Benefit Synthesis

The safety considerations detailed above underscore that no component of the proposed framework is risk-free, and the balance of risks and benefits is likely to vary substantially between individuals depending on baseline cardiovascular and metabolic risk, renal and hepatic function, age, concurrent medications, psychological factors, and personal preference. For an apparently healthy individual with a strong family history of premature cardiovascular disease and early biomarker evidence of dysmetabolism, the potential benefits of this framework—pending confirmatory outcome trials—may reasonably be judged to outweigh its risks under appropriate medical supervision. For a lower-risk individual, the currently unproven nature of hard clinical benefit, combined with the financial cost of CGM, the gastrointestinal burden of acarbose, the rare but serious risk of metformin-associated lactic acidosis, and the physiological demands of fasting, may tip this balance differently. We emphasize that this framework is not a one-size-fits-all recommendation, and individualized, physician-guided risk–benefit discussion, rather than protocolized application, is essential.

## 12. Cost and Accessibility Considerations

The framework proposed in this review involves multiple components with substantial cumulative cost, which is likely to fall largely on the individual, as apparently healthy, non-diabetic adults are unlikely to have insurance coverage for most of these interventions in most healthcare systems. This section provides representative cost estimates and discusses strategies to improve accessibility; formal cost-effectiveness analysis of this framework has not been conducted and is identified as a priority for future research.

### 12.1. Representative Costs of Framework Components

CGM sensors typically cost approximately USD 80–300 per month out of pocket for non-diabetic users, depending on device and subscription model, with individual sensors lasting 10–15 days [56]; the “sprint” model proposed in Phase 1–2 of this framework (repeated 2–4 week periods of CGM use, once or twice annually) would require multiple sensors per year, with cumulative annual cost in the range of several hundred US dollars depending on sprint frequency. Dietitian or health coach consultations are not typically covered by insurance for preventive use in non-diabetic individuals and add further recurring cost, which varies widely by region and provider. DEXA body composition scans, proposed in this framework for biannual monitoring during the fasting integration phase, cost approximately USD 40–200 per scan at dedicated wellness/body-composition facilities in the United States, though hospital-based scans can cost USD 150–400 or more [66] biannual scanning is not covered by insurance when performed for body composition monitoring in healthy individuals rather than for osteoporosis screening, and bioimpedance analysis, while less precise, represents a substantially lower-cost alternative. Metformin and acarbose are both inexpensive, widely available generic medications; however, the associated laboratory monitoring recommended in Section 10 (renal function, vitamin B12) adds recurring cost that varies by healthcare system and is not necessarily covered for off-label, preventive use in non-diabetic individuals.

### 12.2. Insurance Coverage Variability

Coverage for CGM in non-diabetic individuals varies substantially by country and payer. In the United States, CGM is generally covered by insurance only for individuals with diabetes (particularly insulin-treated diabetes); over-the-counter CGM devices intended for non-diabetic wellness use are not covered by most commercial insurance or Medicare, placing the full cost on the individual, though health savings account (HSA) or flexible spending account (FSA) funds may offset some of this burden where available. Coverage policies in other healthcare systems, including those with universal public coverage, are generally similarly restrictive for non-diagnostic, preventive use, though this varies by country and continues to evolve as CGM technology becomes more widely available over-the-counter.

### 12.3. Strategies to Improve Accessibility

Several strategies could reduce the financial burden of this framework and allow more selective, cost-conscious implementation. First, components could be prioritized according to individual risk profile rather than applied uniformly: individuals with a strong family history of premature cardiovascular disease or early biomarker evidence of dysmetabolism may derive proportionally greater expected benefit from the full framework, whereas lower-risk individuals might reasonably limit engagement to lower-cost components (e.g., an initial CGM sprint alone, without progressing to pharmacological augmentation). Second, intermittent “sprint” CGM use, as already proposed in this framework, is inherently less costly than continuous year-round CGM use, and this cost advantage should be made explicit as a rationale for the sprint model, not only a behavioral one. Third, professional (blinded, clinic-based) CGM, in which the device is worn by the patient but data are downloaded and reviewed retrospectively by a clinician rather than viewed in real time, may offer a lower-cost alternative to real-time consumer CGM subscriptions for individuals whose primary goal is periodic metabolic assessment rather than ongoing behavioral feedback, since professional CGM avoids recurring real-time data/subscription fees [67]; this trade-off (lower cost and clinician oversight, versus loss of the real-time behavioral feedback that Section 6.3 identifies as a key mechanism of CGM-guided dietary change) merits explicit consideration when selecting a monitoring approach. Fourth, employer-sponsored wellness programs, which increasingly offer subsidized or free access to CGM and health coaching as part of broader wellness benefits, and enrollment in research studies (which typically provide framework components at no cost to participants), represent additional potential access pathways not dependent on traditional insurance coverage.

### 12.4. Practical Barriers Beyond Cost

Implementation of this framework in real-world clinical practice faces several barriers beyond direct financial cost. Interpreting CGM data, coordinating multi-phase monitoring, and providing structured coaching require clinical time and specific training that may not be readily available, particularly in primary care settings managing high patient volumes. Reliable access to the necessary infrastructure—laboratory monitoring for renal function and vitamin B12, CGM devices, and DEXA or bioimpedance equipment—may be limited in resource-constrained settings independent of cost considerations. Furthermore, the absence of clinical practice guidelines or established reimbursement pathways for preventive, non-diabetic CGM use creates a structural disincentive for health systems to build the infrastructure and training pathways this framework would require, independent of whether individual patients can afford its components. We consider these implementation barriers to be at least as significant as direct cost in many settings, and note that they are unlikely to be resolved until, and unless, the framework accumulates the outcome evidence needed to justify formal guideline incorporation.

## 13. Limitations, Evidence Gaps, and Future Directions

Despite the mechanistic coherence and biological plausibility of the proposed framework, important evidence gaps must be acknowledged:

- Absence of hard cardiovascular endpoint trials: No randomized controlled trial has yet demonstrated that CGM use in non-diabetic individuals reduces myocardial infarction, stroke, or cardiovascular mortality. The mechanistic and surrogate endpoint evidence reviewed here, while compelling, does not substitute for hard outcome data.
- TAME trial pending: The TAME trial results, expected 2026–2027, will provide the first prospective randomized evidence for metformin’s effect on the composite of age-related disease incidence in non-diabetic individuals. Its results will significantly refine the evidence base for low-dose metformin in preventive emerging longevity medicine.
- Acarbose cardiovascular trial limitation: STOP-NIDDM’s cardiovascular analyses were secondary endpoints in a trial powered for diabetes prevention; dedicated cardiovascular outcomes trials with acarbose in non-diabetic individuals are lacking.
- CGM targets for non-diabetics: Optimal CGM metric thresholds for apparently healthy individuals—particularly the “ideal” ΔPPG, CV%, and TIR targets associated with longevity benefit—have not been established in large prospective studies. Reference ranges currently available are derived primarily from diabetes populations.
- Long-term CGM adherence and data fatigue: Psychological effects of continuous glucose awareness—including anxiety, orthorexic tendencies, and data overload—require dedicated study in non-diabetic populations using CGM for preventive purposes.

**Sex Differences in Response to the Proposed Framework**

The framework proposed in this review is presented in a largely sex-agnostic manner, despite biologically plausible reasons to expect sex-specific effects across its components and well-established sex differences in cardiovascular disease risk, presentation, and pathophysiology [68]. This section reviews the available evidence, and its important gaps, for each intervention.

For acarbose, the NIA Interventions Testing Program found median lifespan extension of 22% in male mice versus 5% in female mice, an effect mechanistically attributed to greater postprandial glucose and IGF-1 reduction in males, as female mice maintain lower baseline glucose and IGF-1 levels with correspondingly less room for acarbose-induced reduction [42]. Whether an analogous sex difference exists in humans is unknown; no human study has specifically evaluated sex-stratified cardiovascular or longevity outcomes with acarbose, and this represents a direct evidence gap.

For metformin, sex differences have been described across several domains relevant to this framework. Women are typically prescribed lower metformin doses and more commonly report gastrointestinal side effects than men, while some observational data suggest a lower incidence of cardiovascular events in women taking metformin compared to men taking metformin. Metformin has also been shown to reduce circulating testosterone and estradiol in both sexes via AMPK-mediated modulation of androgen biosynthesis, with uncertain downstream consequences for cardiovascular risk given the established, though sex-differentiated, cardioprotective roles of estrogen in premenopausal women and testosterone in men [69,70] Hormonal status—including menopausal status and use of hormonal contraception or hormone replacement therapy—may further modify glucose metabolism and, plausibly, response to CGM-guided nutrition, fasting, and pharmacological intervention, but this has not been systematically studied in the context of this framework.

For CGM-guided nutrition and fasting protocols, dedicated sex-stratified data are limited. Sex differences in glucose and lipid metabolism, body composition, and hormonal cycling (in premenopausal women) are well established in the broader physiology literature, and it is plausible that these could modify postprandial glucose responses, ketoadaptation kinetics, and tolerability of fasting; however, we identified no studies directly evaluating whether the CGM-guided dietary or fasting components of this framework should differ by sex.

We do not believe the current evidence base is sufficient to support specific sex-differentiated recommendations for any component of this framework, and we explicitly identify this as a critical research gap rather than offering speculative sex-specific protocols. Regarding clinical trials capable of resolving this gap, TAME enrolls approximately 3000 participants and is powered for its composite primary endpoint; it does not appear to have been specifically designed or powered for sex-stratified subgroup analysis, and a null or positive overall result may therefore obscure clinically meaningful differences between sexes. Trials specifically designed with adequate power for pre-specified sex-stratified analysis, or, where this is not feasible, sex-specific trials (an approach already emerging in the metformin/aging field), would be needed to properly address this question. As evidence accumulates, we anticipate that monitoring intervals, dosing strategies, and perhaps the overall risk–benefit calculus of this framework may need to be refined separately for men and women; until then, we recommend that clinicians applying any component of this framework remain attentive to the possibility of sex-differentiated response and adverse effects, without assuming current sex-agnostic dosing and monitoring recommendations are equally optimal for all patients.

As summarized in Table 2, the evidence base for each component of the proposed framework varies substantially in strength and population applicability. Priority future research directions include: a dedicated randomized trial of CGM-guided precision nutrition versus standard dietary advice with composite cardiovascular event endpoints in non-diabetic high-risk individuals; mechanistic studies of acarbose’s IGF-1-lowering and gut microbiome effects in humans; sex-stratified analysis of metformin and acarbose longevity effects in TAME; and prospective registry studies of combined metformin + acarbose + CGM-guided fasting programs with comprehensive longevity biomarker panels.

No formal cost-effectiveness or cost-utility analysis of the framework proposed in this review, or of its individual components, has been conducted in an apparently healthy, non-diabetic population. Given the substantial cumulative cost of the full framework and the current absence of hard cardiovascular outcome data (Table 2), we identify formal cost-effectiveness analysis—ideally conducted alongside or following the prospective outcome studies identified elsewhere in this review as a research priority—as an important and currently unaddressed area for future research, essential for determining whether, and for whom, the framework represents good value relative to its cost.

A personal perspective illustrating the clinical motivation behind this review is presented in Box 1. This account is a single, unblinded personal observation (*n* = 1), offered as motivating context only; it does not constitute clinical evidence and should not be read as demonstrating efficacy.

Box 1 **Author Perspective: From Clinical Observer to Metabolic Patient.**

The impetus for this review is not solely academic. The first author is a board-certified cardiologist who has simultaneously occupied the role of high-risk patient—a dual position that has profoundly shaped the clinical and scientific framing of the evidence presented here.

The family history that informs this work began with a father whose cardiovascular trajectory exemplifies the natural history of uncontrolled dysmetabolism: decades of hypertension, type 2 diabetes, and progressive coronary artery disease requiring repeated revascularization—a lifetime of compounding metabolic injury culminating in a phenotype this article is dedicated to preventing. Witnessing this trajectory firsthand as both a daughter and a cardiologist created a professional and personal imperative to apply the most rigorous preventive tools available—not to others alone, but to oneself.

By her early 30s, the author’s own cardiovascular risk profile was already clinically significant, with hypertension, dyslipidemia, and an adverse genetic risk profile placing her in the moderate-to-high risk category—a striking finding for a woman in her fourth decade of life. A first pregnancy brought these risks into sharp relief, complicated by gestational hypertension and gestational diabetes requiring insulin, which first introduced the author to the behavioral and informational power of real-time glucose monitoring. The postpartum metabolic picture—metabolic syndrome, prediabetes, persistent hypertension, dyslipidemia—reflected precisely the constellation of risk factors this review addresses.

What followed was a structured, CGM-guided intervention applied with the same rigor the author would advocate for in clinical practice. Within six months, this approach was associated with normalization of HbA1c, resolution of insulin resistance, normalization of the lipid profile, and reclassification from high to low cardiovascular risk. The CGM data themselves served as a day-by-day behavioral learning instrument, making visible the metabolic consequences of specific food choices, meal sequences, and activity patterns in a way abstract dietary counseling had never achieved.

This personal experience does not constitute clinical evidence—it is offered here as context, not proof. The authors are aware that physician self-experimentation carries inherent cognitive biases and cannot substitute for the randomized controlled trials identified as research priorities in the Limitations section. Nevertheless, the convergence of mechanistic evidence, surrogate endpoint data, and lived metabolic experience forms the motivating substrate for the synthesis presented in this review.

## 14. Conclusions

The convergence of CGM technology, fasting biology, and longevity pharmacology represents a promising research direction for preventive cardiology in apparently healthy individuals, grounded in a coherent mechanistic rationale rather than in established clinical outcome evidence. CGM reveals glycemic variability that is not captured by conventional screening and that may serve as both a risk marker and a modifiable target, although validated non-diabetic thresholds do not yet exist. Personalized nutrition guided by CGM data offers a plausible, low-risk means of translating these observations into behavioral change, though hard cardiovascular outcome data are lacking. Intermittent and prolonged fasting activate cellular programs—autophagy, reduced IGF-1 and mTORC1 signaling—that are associated with extended healthy lifespan in model organisms and favorable short-term risk-factor changes in small human studies, but corresponding cardiovascular outcome trials in healthy non-diabetic adults have not been conducted. Low-dose metformin and acarbose each have biologically plausible mechanisms and supportive observational, preclinical, or secondary-analysis data, but neither has been shown in a dedicated randomized trial to reduce cardiovascular events or extend lifespan in apparently healthy, non-diabetic adults; the TAME trial, once complete, will be an important step toward addressing this gap for metformin.

These interventions share a proposed mechanistic coherence, converging on the attenuation of overactive nutrient-sensing pathways (insulin/IGF-1, mTORC1) implicated in both cardiovascular disease and biological aging. CGM offers a personalized biofeedback layer that could, in principle, make these interventions more measurable and motivating; whether this translates into improved clinical outcomes remains to be tested.

We present the four-phase framework described in this review as a structured, testable research model rather than a validated clinical protocol. Its individual components vary substantially in evidence strength and carry safety considerations that require medical supervision rather than self-directed implementation. Before any component of this framework can be recommended for clinical use in apparently healthy, non-diabetic adults, prospective studies—ideally randomized and powered for cardiovascular and safety endpoints—are needed to establish efficacy, safety, feasibility, and long-term adherence. We view this synthesis as a hypothesis-generating contribution intended to help prioritize and structure that future research, rather than as a basis for immediate clinical implementation.

## Figures and Tables

**Table 1:** CGM metrics for non-diabetic individuals: definitions, proposed reference targets (with evidence basis), and cardiovascular/longevity significance. Targets are exploratory and largely extrapolated or author-proposed; none are established clinical or regulatory standards in non-diabetic populations.

CGM Metric	Definition	Target (Non-Diabetic)	CV/Longevity Significance	Evidence Basis
Time in Range (TIR) [17]	% readings within 3.9–7.8 mmol/L	>97%	TIR < 90% indicates clinically relevant dysglycemia; inversely correlated with subclinical atherosclerosis markers	Extrapolated from international consensus TIR targets developed for diabetic/prediabetic populations [17]; not separately validated in healthy non-diabetic adults
Time Above Range (TAR) [17]	% readings > 7.8 mmol/L	<3–5%	Captures cumulative postprandial glycemic burden; each 1% increase in TAR associated with greater endothelial oxidative stress and AGE accumulation	Extrapolated from diabetes-derived consensus thresholds; author-proposed adaptation for non-diabetic use
Coefficient of Variation (CV%) [17]	SD of glucose/mean glucose × 100	<20%	CV% > 36% independently associated with hypoglycaemia risk and adverse CV outcomes; oscillating glucose more damaging to endothelium than sustained mild hyperglycaemia	Adapted from diabetes CV% thresholds; the “<20%, longevity-optimal” framing is author-proposed and not independently validated |
Mean Amplitude of Glycemic Excursions (MAGE) [18]	Mean of glucose excursions exceeding 1 SD from mean	Minimise; lower = better	Lower MAGE correlates with reduced oxidative stress markers and carotid IMT; strongly predicts MACE in ACS patients independent of diabetic status	Derived from observational/mechanistic association studies (oxidative stress, carotid IMT, MACE prediction; no validated non-diabetic target value established
Incremental Postprandial Rise (ΔPPG) [17]	Peak − pre-meal glucose within 2 h	<1.7–2.0 mmol/L	Direct target for dietary personalisation; ΔPPG reduction by meal sequencing 20–40%; by post-meal walking 15–30%; drives NF-κB-mediated endothelial inflammation when elevated	Author-proposed threshold based on mechanistic and observational data linking ΔPPG magnitude to endothelial inflammation; not a validated clinical cutoff

Abbreviations: AGE, advanced glycation end-product; ACS, acute coronary syndrome; CGM, continuous glucose monitoring; CV, cardiovascular; IMT, intima-media thickness; MACE, major adverse cardiovascular events; SD, standard deviation; TAR, time above range; TIR, time in range.

**Table 2:** Summary hierarchy of evidence for major interventions discussed in this review, by population, study design, endpoints, evidence strength, limitations, and applicability to apparently healthy, non-diabetic adults.

Intervention	Population Studied	Study Type	Endpoints Assessed	Strength of Evidence	Key Limitations	Applicability to Non-Diabetic Adults
CGM (as monitoring tool) [12,14,16,17]	Diabetic, prediabetic; limited non-diabetic cohorts	Observational; device validation studies; small mechanistic studies in non-diabetics	Glycemic metrics (TIR, TAR, CV%, MAGE, ΔPPG); surrogate vascular markers (endothelial function, IMT) in some studies	Moderate for glycemic pattern detection; low for CV outcome prediction in non-diabetics	Few dedicated non-diabetic outcome studies; targets extrapolated from diabetic populations	High for glycemic self-monitoring/behavioral feedback; unestablished for driving hard CV outcomes
CGM-guided dietary personalization [3,27]	Mixed diabetic/non-diabetic cohorts	Cohort studies; short-term randomized trials	Postprandial glucose response, glycemic variability	Moderate for short-term glycemic improvement; low for long-term CV/longevity outcomes	Short follow-up; heterogeneous protocols; no hard-endpoint trials	Plausible and low-risk; benefit on hard outcomes unproven
Intermittent fasting/time-restricted eating [6,18,19,20]	Overweight/obese non-diabetic adults (mostly short-term)	Small RCTs, pilot studies	Weight, fasting insulin, LDL, inflammatory markers, blood pressure	Moderate for short-term cardiometabolic risk factor improvement; low for hard CV/longevity endpoints	Short duration (weeks); small samples; heterogeneous protocols	Reasonably applicable; risk factor benefit supported, outcome benefit unproven
Prolonged fasting (24–120 h) [13,21,22,23,24]	Small human cohorts; extensive preclinical/mechanistic data	Mechanistic human studies; preclinical (rodent) studies	Autophagy markers, IGF-1, ketone body kinetics	Strong mechanistic/preclinical; very limited human clinical/outcome data	Feasibility, safety, and adherence not established at scale; no CV outcome trials in healthy adults	Mechanistically plausible; safety/efficacy in healthy non-diabetic adults unestablished, requires supervision
Metformin (low-dose, non-diabetic use) [35,36,37,38,39]	Type 2 diabetic cohorts (observational); non-diabetic older adults (ongoing trial, TAME); model organisms (ITP)	Observational cohort (diabetic); preclinical (mouse); RCT ongoing/pending (TAME)	All-cause mortality (observational, diabetic); lifespan (mouse); composite age-related disease (TAME, pending)	Moderate-to-strong in diabetic populations (observational); weak/preliminary in non-diabetics pending TAME	No completed RCT in non-diabetic humans; observational survival association subject to healthy user effect, indication bias, and immortal time bias; TAME results pending (~2026–2027)	Mechanistically plausible; human longevity/CV benefit in non-diabetics not yet established
Acarbose [40,42,43,44,45,46]	Individuals with impaired glucose tolerance (STOP-NIDDM); type 2 diabetic cohorts (meta-analysis); model organisms (ITP)	RCT with secondary CV analysis (STOP-NIDDM); meta-analysis (diabetic); preclinical (mouse)	Diabetes incidence (primary); CV events, MI (secondary/meta-analysis); lifespan (mouse)	Moderate for glycemic/diabetes prevention in IGT; weak for CV benefit—initial secondary-analysis signal (STOP-NIDDM) not replicated in adequately powered trial (ACE); strong preclinical lifespan signal	STOP-NIDDM CV finding not powered/underpowered and not replicated in the larger ACE trial (HR 0.98, CI 0.86–1.11); no trial in normoglycemic healthy adults	Mechanistically plausible for postprandial spike reduction; CV/longevity benefit in healthy non-diabetics unestablished

## Data Availability

No new data were created or analyzed in this study. Data sharing is not applicable to this article.

## Abbreviations

The following abbreviations are used in this manuscript:

AGE	Advanced glycation endproduct
AIx	Augmentation index
ADF	Alternate-day fasting
AMPK	AMP-activated protein kinase
CALERIE	Comprehensive Assessment of Long-term Effects of Reducing Intake of Energy
CGM	Continuous glucose monitoring
CRP	C-reactive protein
CVD	Cardiovascular disease
DEXA	Dual-energy X-ray absorptiometry
ΔPPG	Incremental postprandial glucose excursion
eGFR	Estimated glomerular filtration rate
eNOS	Endothelial nitric oxide synthase
FOXO	Forkhead box O transcription factor
GLP-1	Glucagon-like peptide-1
GLUT4	Glucose transporter type 4
HDAC	Histone deacetylase
HbA1c	Glycated haemoglobin
HCAR2	Hydroxycarboxylic acid receptor 2
HFpEF	Heart failure with preserved ejection fraction
HIIT	High-intensity interval training
HOMA-IR	Homeostatic model assessment of insulin resistance
hsCRP	High-sensitivity C-reactive protein
ICAM-1	Intercellular adhesion molecule-1
IF	Intermittent fasting
IGF-1	Insulin-like growth factor-1
IL-6	Interleukin-6
ITP	Interventions Testing Program
LDL	Low-density lipoprotein
MACE	Major adverse cardiovascular events
MAGE	Mean amplitude of glycemic excursions
MARD	Mean absolute relative difference
mTORC1	Mechanistic target of rapamycin complex 1
NF-κB	Nuclear factor kappa B
NIA	National Institute on Aging
NLRP3	NOD-, LRR- and pyrin domain-containing protein 3
RAGE	Receptor for advanced glycation endproducts
SCFA	Short-chain fatty acid
SIRT1	Sirtuin-1
SIRT3	Sirtuin-3
SREBP1	Sterol regulatory element-binding protein 1
STOP-NIDDM	Study to Prevent Non-Insulin-Dependent Diabetes Mellitus
TAR	Time above range
TAME	Targeting Aging with Metformin
TIR	Time in range
TNF-α	Tumour necrosis factor alpha
TRE	Time-restricted eating
UKPDS	UK Prospective Diabetes Study
VCAM-1	Vascular cell adhesion molecule-1
β-OHB	Beta-hydroxybutyrate
